# Mapping the Structure-Function Relationship in Glaucoma and Healthy Patients Measured with Spectralis OCT and Humphrey Perimetry

**DOI:** 10.1155/2018/1345409

**Published:** 2018-04-17

**Authors:** Laia Jaumandreu, Francisco J. Muñoz–Negrete, Noelia Oblanca, Gema Rebolleda

**Affiliations:** ^1^Ophthalmology Service, University Hospital Ramón y Cajal, School of Medicine and Health Science, University of Alcalá, IRYCIS, Hospital Ramón y Cajal, Ctra. Colmenar Viejo km. 9100, 28034 Madrid, Spain; ^2^Ophthalmology Service, University Hospital Ramón y Cajal, IRYCIS, Hospital Ramón y Cajal, Ctra. Colmenar Viejo km. 9100, 28034 Madrid, Spain

## Abstract

**Purpose:**

To study the structure-function relationship in glaucoma and healthy patients assessed with Spectralis OCT and Humphrey perimetry using new statistical approaches.

**Materials and Methods:**

Eighty-five eyes were prospectively selected and divided into 2 groups: glaucoma (44) and healthy patients (41). Three different statistical approaches were carried out: (1) factor analysis of the threshold sensitivities (dB) (automated perimetry) and the macular thickness (*μ*m) (Spectralis OCT), subsequently applying Pearson's correlation to the obtained regions, (2) nonparametric regression analysis relating the values in each pair of regions that showed significant correlation, and (3) nonparametric spatial regressions using three models designed for the purpose of this study.

**Results:**

In the glaucoma group, a map that relates structural and functional damage was drawn. The strongest correlation with visual fields was observed in the peripheral nasal region of both superior and inferior hemigrids (*r* = 0.602 and *r* = 0.458, resp.). The estimated functions obtained with the nonparametric regressions provided the mean sensitivity that corresponds to each given macular thickness. These functions allowed for accurate characterization of the structure-function relationship.

**Conclusions:**

Both maps and point-to-point functions obtained linking structure and function damage contribute to a better understanding of this relationship and may help in the future to improve glaucoma diagnosis.

## 1. Introduction

Perimetry is classically considered the “gold standard” for glaucoma diagnosis, but a significant loss of retinal ganglion cells (25–30%) occurs before any of the typical glaucomatous visual field (VF) defects are detected [1]. Several studies have found that the combination of data obtained from structural and functional tests improves the diagnostic capability of each of these tests individually [2, 3]. As a result, researchers are now focusing on exploring in depth the relationship between structure and function in glaucoma.

In 1998, Zeimer et al. [4] first suggested imaging of the macula as a potential structure for the diagnosis of glaucoma, and it has now been widely demonstrated that thinning of the macula occurs in glaucoma as a result of the retinal ganglion cells (RGC) loss typical of this pathology [5, 6]. Several authors have studied the correlation between perimetry and macular thickness [7–13]. Most of them used simple correlation and linear regression between global indices and by sectors. We believe that this approach falls short in terms of providing an accurate explanation of the structure-function relationship. In this study, we take one more step making an analysis by regions and point to point using the macular grid given by the Spectralis OCT and novel statistical approaches. These approaches, some of which had not been employed previously in the field, may contribute to a better understanding of the relationship.

## 2. Materials and Methods

### 2.1. Subjects and Experimental Design

The research protocol followed the tenets of the Declaration of Helsinki and was approved by the ethical committee of University Hospital Ramón y Cajal, Madrid, Spain. Informed consent was obtained from each participant before enrollment after explanation of the nature and possible consequences of the study. A cohort of patients was prospectively selected according to the following inclusion criteria: between 18 and 80 years of age, best corrected visual acuity > 20/40 (Snellen) in the study eye, refractive error within ±5.00 dioptres equivalent sphere and ±2.00 dioptres astigmatism, and transparent ocular structures: crystalline lens opacity < 1 in LOCS III (Lens Opacities Classification System) [14] and availability and collaboration to perform protocol exploratory tests. Patients with any kind of retinopathy, who had previously undergone ocular surgery except phacoemulsification without complications, with a history of neuroophthalmic disorder, ocular malformations, angle or optic nerve anomalies, or who had any serious disease or current use of a medication that could affect visual field sensitivity, were excluded from the study.

The cohort of patients was divided into 2 groups: patients with glaucoma or “cases” and healthy patients or “controls.” The glaucoma subjects had to meet two diagnosis criteria: (1) glaucomatous appearance of the optic disc evaluated by a glaucoma specialist, defined as focal or diffuse neuroretinal rim narrowing with concentric enlargement of the optic cup, localized notching or both [15] and (2) perimetric criteria for glaucoma: glaucoma Hemifield Test (GHT) results outside normal limits, a pattern standard deviation (PSD) with a *P* value < 5%, or a cluster of three or more nonedge points on the pattern deviation plot in a single hemifield with *P* values < 5%, one of which must have a *P* value < 1%.

All the patients in the study underwent the following series of tests: general anamnesis, basic eye examination, optical coherence tomography with Spectralis OCT® (Heidelberg Engineering, Heidelberg, Germany), and at least two reliable automated conventional perimetry tests of both eyes with Humphrey visual field analyzer (Carl Zeiss Meditec, Dublin, California, USA). The perimetric test was performed with SITA (Swedish interactive threshold algorithm) standard 24-2 strategy. The following reliability criteria were adopted: fixation losses, false-positive rate, and false-negative rate less than 20% [16, 17]. The last reliable perimetry test obtained was used in this study to minimize the learning effect and only reproducible visual field defects were taken into account [18, 19]. The protocol selected on the Spectralis OCT was the posterior pole asymmetry analysis which measures retinal thickness in the posterior pole using 61 lines (30° × 25° OCT volume scan) for each eye in a central 20 degree area. Only the OCT scans with a signal equal to or higher than 24 that were adequately centred on the fovea and had no eye movement or blinking artefacts were considered.

### 2.2. Statistical Analysis

Descriptive and correlation statistical analyses were carried out using SPSS statistical software for Windows (version 20.0, IBM-SPSS, Chicago, Illinois, USA). Nonparametric regression analyses were coded in GAUSS (9.0 version, light). In order to facilitate analysis, all data were converted to left eye data. A statistical significance of *P* < 0.05 was required for all comparisons.

In order to study differences between the two groups, the following variables were compared: age, sex, laterality, test time, as well as mean and standard deviation of visual field index (VFI), mean deviation (MD), and average thickness total, superior, and inferior (ATT, ATS, ATI). The Kolmogorov-Smirnov test was used to assess the normality of distribution.

A factor analysis, principal component type, was carried out as a first approach to the study of the structure-function relationship. This was based on a previous analysis described by Ferreras et al. in 2008 [16]. We took as random variables: mean threshold sensitivities (dB) of the automated perimetry (excluding from the analysis those corresponding to the blind spot) and mean macular thickness (*μ*m) calculated for each 3° × 3° square of the macular grid. Separate analyses were carried out for each group—glaucoma and healthy patients—and for each hemifield and hemigrid individually (assuming that the superior and inferior hemifields and hemigrids are anatomically distinct). Each variable was assigned to a factor or principal component, obtaining as a result interrelated groups of variables and, thus, determining the regions. Pearson's correlation was subsequently applied to these regions (superior hemifield with inferior hemigrid and inferior hemifield with superior hemigrid) in both groups.

**Figure 1:**
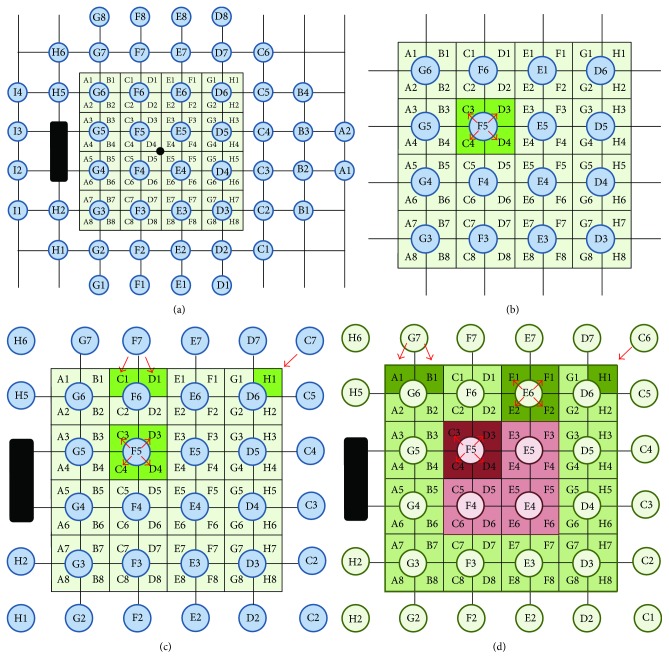
(a) Spatial correspondence between the measuring points of threshold sensitivity in the automatized perimetry (Humphrey, 24-2 program) and the macular thickness map (Spectralis OCT). (b) Graphic scheme of the first nonparametric spatial regression model. The measurements of threshold sensitivity in each of the 16 central points of the visual field are regressed on the macular thickness of the four surrounding areas. (c) Graphic scheme of the second nonparametric spatial regression model. The measurements of threshold sensitivity in each of the 34 central points of the visual field are regressed on the macular thickness of either the four surrounding areas for the 16 central points or the closest one or two areas (depending on the position) for the 18 most eccentric points. (d) Graphic scheme of the third nonparametric spatial regression model. Two separate regressions were performed: the measurements of threshold sensitivity in each of the 4 central points of the visual field on the macular thickness of the four surrounding areas (red) and the measurements in each of the other 30 points (green) on the 4, 2, or 1 (depending on the position) closest areas.

The second approach was nonparametric regression analysis. Nonparametric regression analysis allows the study of the relationship between two variables, *x* and *y*, when this relationship is given by an unknown function that is determined as *m*(*x*) plus the prediction errors *u*(*y* = *m*(*x*) + *u*). We used the Nadaraya-Watson estimator with standard normal kernel [20]. Threshold sensitivity (dB) was established as the dependent variable (*y*) and macular thickness (*μ*m) as the independent variable (*x*) in all cases. Two types of regression were applied. First, nonparametric regressions were calculated for each pair of zones or regions, obtained through factor analysis, that showed significant correlation according to Pearson's correlation coefficient in the glaucoma group. Then, several nonparametric spatial regressions were calculated using three models designed by our team for the purpose of this study. The models are based on the spatial correspondence between the measuring points of threshold sensitivity in the automated perimetry and the macular thickness map, as shown in Figure 1(a), assuming the same principles of spatial correspondence than other authors [12, 13]. The study was carried out separately for the two groups (glaucoma and control). The three nonparametric spatial regression models used are summarized and explained in Figures 1(b)–1(c).

## 3. Results

A total of 85 eyes of 58 Caucasian patients selected according to the inclusion criteria were included in this study (44 glaucoma; 41 control).  Table 1 summarizes global descriptive statistics obtained in both groups. The patients in the glaucoma group had a mean MD of −7.73 ± 5.58 dB. All the macular thickness indices analysed (ATT, ATS, and ATI) showed statistically significant differences between both groups, and their values were always lower in the glaucoma cohort. The study also revealed a significant difference between the mean macular thickness of the superior and inferior hemigrids in the glaucoma group, while the control group did not show any significant difference between them.

### 3.1. Factor Analysis and Pearson's Correlation Coefficient between the Obtained Regions

The measure of sampling adequacy (MSA), KMO (Kaiser-Meyer-Olkin) measure, was greater than 0.6 in all cases and the total variance explained by the selected components was >80%.

**Figure 2:**
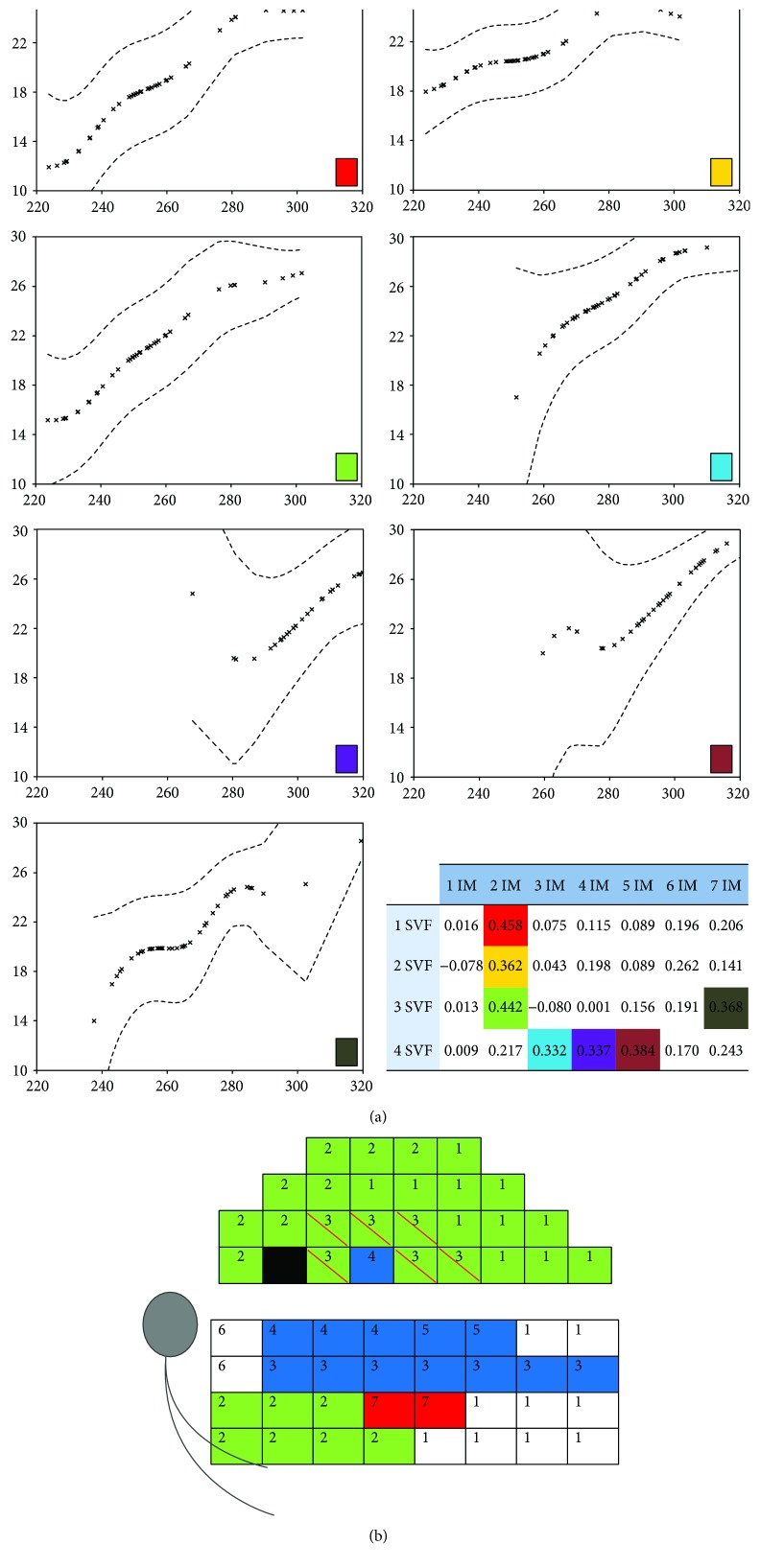
(a) Nonparametric regression between the regions of the superior hemifield (1–4 superior visual field, SVF) and inferior hemigrid (1–7 inferior macula, IM) obtained through factor analysis and that showed significant correlation according to Pearson's correlation coefficient (shown at the bottom right of the graphics) in the glaucoma group. Values of threshold sensitivity (decibels) are represented in the *y*-axis and values of macular thickness (microns) in the *x*-axis. (b) Graphic scheme of the Pearson correlations between the regions obtained through factor analysis of the superior hemifield (1–4) and factor analysis of the inferior hemigrid (1–7) in glaucoma cases. Regions that showed significant correlations are represented with the same color.

**Figure 3:**
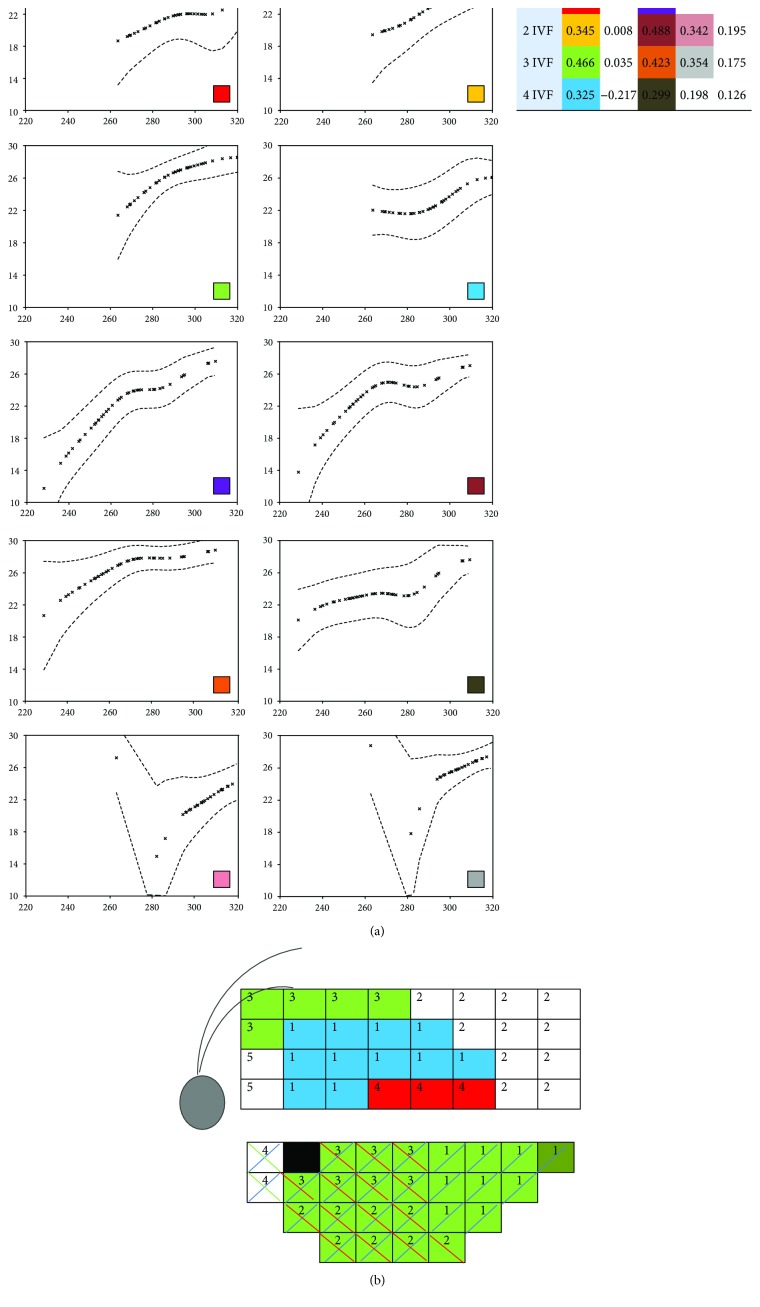
(a) Nonparametric regression between the regions of the inferior hemifield (1–4 inferior visual field, IVF) and superior hemigrid (1–5 superior macula, SM) obtained through factor analysis and that showed significant correlation according to Pearson's correlation coefficient (shown at the top right of the graphics) in the glaucoma group. Values of threshold sensitivity (decibels) are represented in the *y*-axis and values of macular thickness (microns) in the *x*-axis. (b) Graphic scheme of the Pearson correlations between the regions obtained though factor analysis of the inferior hemifield (1–4) and factor analysis of the superior hemigrid (1–5) in glaucoma cases. Regions that showed significant correlations are represented with the same color. Correlations with *P* < 0.05 are represented by colored thin strips.

In the glaucoma group, the factor analysis determined 4 regions or factors in both superior and inferior hemifields of the automatized perimetry, and 5 regions in the superior hemigrid and 7 in the inferior hemigrid of the Spectralis OCT macular grid (Figures 2(b) and 3(b)).

In contrast, in the control group, the factor analysis determined 3 regions or factors in the superior hemifield and 5 in the inferior hemifield of the automated perimetry, and 3 regions in the superior hemigrid and 5 regions in the inferior hemigrid of the Spectralis OCT macular grid.

Pearson's correlation coefficients showed statistically significant differences between the associated anatomical-functional regions in the glaucoma group (Figures 2(a), 2(b), 3(a), and 3(b)). The strongest correlation with visual field was observed in the peripheral nasal region of both macular hemigrids (superior *r* = 0.602; inferior *r* = 0.458), while the temporal peripapillary and temporal peripheral regions did not show any significant correlation. In contrast, no significant correlation was found in the control group.

### 3.2. Nonparametric Regression between the Regions Obtained through Factor Analysis

Figures 2(a) and 3(a) depict the regression curves between regions that showed significant correlation according to Pearson's correlation coefficient in the glaucoma group. These curves represent the mean threshold sensitivity values for each thickness value of the macular thickness map of each respective region. Only the mean threshold sensitivity values with corresponding thickness data available were plotted. Since no significant correlations between the regions were observed in the control group, we omit regression for those data. All the regression curves showed a similar pattern: an area of ascending slope (more or less steeper depending on the pair under study) that takes a shape similar to a linear function where the greater the macular thickness is, the greater the sensitivity is, and another area with a slope that is close to zero in which the threshold sensitivity is maintained more or less constant despite the increase in macular thickness. The functions showing greater linear correlation and steeper slopes corresponded to the correlation studies between the pairs of regions that showed a stronger linear relationship (higher Pearson's correlation coefficient). The nonparametric regression curves obtained for the paracentral and peripheral regions showed similar characteristics among them. This also happened with the curves obtained for the central regions.

### 3.3. Nonparametric Spatial Regression

**Figure 4:**
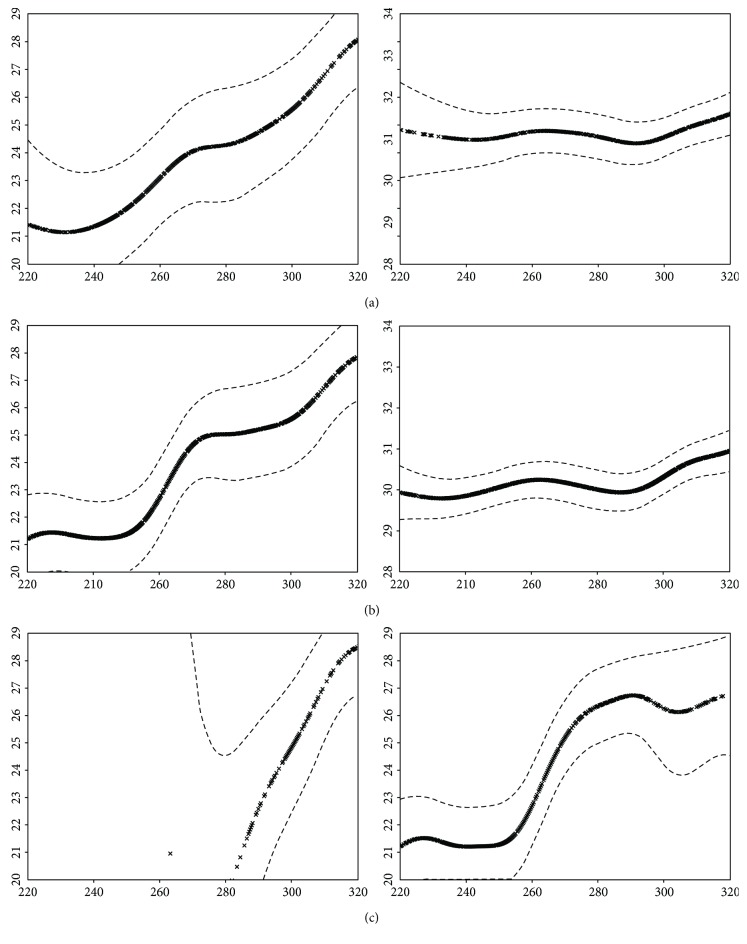
(a) Regression curves obtained with the application of the first model in the glaucoma (left) and control (right) groups. Dashed lines represent 90% confidence intervals. Values of threshold sensitivity (decibels) are represented in the *y*-axis and values of macular thickness (microns) in the *x*-axis. (b) Regression curves obtained with the application of the second model in the glaucoma (left) and control (right) groups. (c) Regression curves for centre (left) and periphery (right), obtained with the application of the third model in the glaucoma group.

The first model used studied the function-structure relationship between the 16 central points in the visual field and the complete macular grid (Figure 1(b)). An approximately linear ascending relationship can be observed in the glaucoma group where sensitivity (dB) increases alongside macular thickness (*μ*m), although there are two sections of the curve in which the slope is close to zero. One is located between 220 and 240 *μ*m and the other at the halfway point between 270 and 290 *μ*m (Figure 4(a)).

For the second model, the 18 points in the visual field located around the previous 16 were added to the regression curve, so that the model studied the function-structure relationship between 34 central points in the visual field and the complete macular grid (Figure 1(c)). The graph obtained for the glaucoma group (Figure 4(b)) was very similar to that of the first regression model, therefore confirming the shape of the relationship. An ascending relationship with, once again, two sections in which the slope is close to zero can be observed. These two sections appear at the same point as in the first model (220 and 270 *μ*m) but stretch slightly further in both cases.

The third model divided the data into two subgroups: the peripheral and the central macular areas (Figure 1(d)). Independent regression curves were drawn for each subgroup. The peripheral area in the glaucoma group showed an ascending relationship with two horizontal tails, while the central area showed only one ascending section (Figure 4(c)).

None of these three models revealed any significant correlation between structure and function in the control group (Figures 4(a) and 4(b)).

## 4. Discussion

We successfully drew a map linking functional and structural damage in the glaucoma group using Humphrey perimetry and the posterior pole asymmetry analysis of the Spectralis OCT regions, obtained completely from an objective analysis, and several functions that provide the mean sensitivity that would correspond to each given macular thickness in glaucoma patients.

In contrast, consistently with previously studies, we have not found any relevant correlation between structure and function in healthy patients [17, 21, 22].

Factor analysis is a data reduction tool for statistical analysis that summarizes data supplied by a group of variables into a smaller set of representative factors. Ferreras et al. [16] first suggested the clustering of threshold points in VF testing using this technique. The advantage of establishing groups of threshold points in this way is that the cluster is not subject to anatomical knowledge of the RNFL or to any preconceived ideas about the relationships in the VF. Another advantage of carrying out factor analysis is that both direct and indirect relationships between the various threshold points are taken into consideration.

Some previous studies on the structure-function relationship maintain that the units of measurement in both must be the same (linear or logarithmic) [23]. However, we performed the study both in decibels (dB) and microns (*μ*m), and in apostilb (asb) and *μ*m, obtaining equivalent outcomes. In this paper, we present the results maintaining the values of the perimetric variables in dB and the macular thickness values in *μ*m because this allows for a more intuitive interpretation of the data and the relationships.

Our VF maps, although with some differences, are overall similar to those obtained in previous studies in which factor analysis of the visual field was carried out [16, 24]. As far as we know, such an analysis has not been previously carried out for the macular hemigrids. The regions obtained were different in the two groups analysed and asymmetrical between hemifields and hemigrids. However, a general similar pattern was observed in all of them.

All the global parameters used to measure total macular thickness (ATT, ATS, and ATI) were significantly lower in the glaucoma group than in the control group. This agrees with previous studies [25–28] and demonstrates the thinning of the macula in glaucoma patients. Mathers et al. [9], also using the Spectralis OCT posterior pole asymmetry analysis, found that patients with a mean macular thickness greater than 300 *μ*m showed practically normal VF. In our study, the mean total macular thickness in the control group was 290.95 *μ*m.

Although most of previous studies agree that the inferior hemimacula shows the strongest correlation with the VF, they do not agree that the peripheral nasal region has the strongest correlation [21, 29, 30]. Only Kim et al. [12], that analysed the point-wise relationship between VF and macular retinal thickness with the posterior pole asymmetry analysis, found that it was stronger in the central and nasal test points (range *r* = 0.14–0.38). Rolle et al. [13], also with this protocol, obtained the strongest correlation in nasal inferior (*r* = 0.55) and temporal inferior quadrant (*r* = 0.57). In the protocols to map the thickness of the macular and the inner retinal layers heretofore used, the nasal and papillomacular bundles are assessed in the same sector. It has been established that the papillomacular bundle is only affected in the advanced stages of glaucoma [28, 31, 32]. This fact may have caused masking of stronger correlations in the nasal peripheral sector in the results obtained until now. In our study, the regions established through factor analysis distinguish between the temporal peripapillary and peripheral nasal regions, and we analyse them separately. The results obtained showed that no significant correlation existed between both hemimacular of the peripapillary temporal region (through which the fibers of the papillomacular bundle would enter) and any of the field regions. By contrast, the peripheral nasal region showed the strongest correlation with VF.

Further studies with protocols to map both regions separately, in different populations and with larger samples, are required in order to confirm whether this region indeed presents better structure-function correlation than the temporal region.

The application of nonparametric regression analysis between each pair of areas that had shown a significant Pearson correlation allowed for the confirmation, quantification, and accurate detection of the characteristics of this relationship. All pairs displayed similar characteristics, especially in the relationships between peripheral and central regions, although some distinctive features were also observed. The shape of the curve resembles the “hockey” or “broken stick” statistical model used by several authors to determine the cut-off point at which peripapillary RNFL thickness begins to show correlation with visual field defects [17, 33, 34].

Nonparametric spatial regression is the third approach to assess structure-function correlation that is suggested in this study. This approach moves away from the principles of classic statistical analysis towards more recent trends and innovations in the field, and it allows for the adoption of a novel perspective to the study of the structure-function relationship. The three nonparametric spatial regression models applied offer an alternative approach that bypasses the need to factor analysis and estimates on average an unknown relationship that is presupposed to exist in all the patients. The estimated function provides the mean sensitivity that would correspond to each given macular thickness, both in glaucoma patients and in healthy ones.

The earliest studies on the structure-function relationship were carried out using statistical models that in one or another way tried to explain this relationship in a linear manner [23]. However, researchers found that good linear correlation only appeared in certain intervals but not throughout, and that often the relationship obtained fitted better a curve rather than a line. For example, Kim et al. [12] found that the global structure-function association was better explained with a quadratic regression model than the linear regression. Garway-Heath et al. [35, 36] suggested that this trend could be the consequence of measuring the dimensions using units that had very different characteristics but this does not fully explain the relationships obtained to the present. Gonzalez-Hernandez et al. [22] correlating the standard automated perimetry mean sensitivity and the global mean RNFL throughout different stages of glaucoma found that the curvilinear relationship between the morphologic and perimetric results may be due to the wide variability in normal morphology and limitations in the dynamic range of the morphologic tests in cases with moderate and severe defects.

There are several facts that suggest a priori that the structure-function relationship at macular level may adjust better to a curvilinear model than to linear regression.

On the one hand, it has been demonstrated that there is a “floor effect” or residual thickness, which has been studied extensively in perimetry-peripapillary RNFL correlations. This concept can also be applied to the measurement of macular thickness if understood from a wider perspective.

On the other hand, several studies suggest that structural defects precede alterations in the visual field [37, 38]. It is also important to consider that early diagnosis depends on the resolution of the system used. Over the past ten years, OCT models have significantly improved in resolution power and accuracy of measurements, while automated perimetry has not changed significantly in the last twenty years. If we accept a linear correlation model for the relationship between structure and function, we would be suggesting that neither parameter affects the other earlier than the other, but rather that the effect is simultaneous.

Other authors [39, 40] have also described linear relationships with varying slope as retinal eccentricity increases, which could point to the appearance of nonlinear relationships in the global analysis of the data.

In conclusion, none of the statistical models currently available fits or can explain thoroughly the characteristics of the relationship between structure and function in glaucoma.

The nonparametric spatial regression models suggested in this study try to resolve many of the weakness in previous studies. Their greatest advantage is that no preestablished functional form is imposed on the data, therefore allowing for the data to determine and draw both linear and nonlinear relationships. They also allow for the use of the most commonly used units of measurement and enable the use of all the values obtained in the analysis of both tests. The relationships can be spatially mapped in order to explore correspondences in more depth than through the application of global parameters or point averages in specific regions. We obtain a function than provides the mean sensitivity that would correspond to each given macular thickness in glaucoma patients improving our understanding of the structure-function relationship.

Although carrying out the correlation study between structure and function using the posterior pole asymmetry analysis provided with Spectralis OCT has several advantages, it is limited by the fact that it can only measure the total thickness of the macula and not the specific layers that are usually more affected in glaucoma (retinal ganglion cell layer). This can increase data noise and the results can also be affected by other eye conditions. This protocol adjusts better to the 24-2 pattern than other systems and we chose this pattern because it is the most often used in clinic in the diagnosis and monitoring of glaucoma; however, it is possible that some relevant information was lost, especially in the centremost points in the visual field [41].

Another limitation of this study is that statistically significant differences in age were found between the two groups. Patients were obtained prospectively and included in one or another group according to the inclusion criteria. The fact that glaucoma is a more frequent pathology in elderly people and that for being included in the control group it was required to lack any other ophthalmic pathology explains this difference. Previous studies have shown that RNFL thickness and visual field sensitivity decrease with age [42–45]. This could induce some bias when comparing the results between both groups. However, because our research focuses on studying separately the correlation between structure and function in glaucomatous or healthy eyes, we think this difference does not imply a distortion in the interpretation of the data.

## 5. Conclusions

This study explores the relationship between structure and function in glaucoma, using novel statistical approaches, and some of which had not been employed previously in this field. The VF test point measures show significant correlation with the corresponding macular thickness points, varying across different regions. The map linking structural damage and functional damage and the corresponding point-to-point functions can be used in the future to improve glaucoma diagnosis and be an additional structural assessment tool.

Further work is necessary in order to confirm these results. The development of more powerful image resolution and better analytical algorithms as well as better functional tests will eventually allow for more accuracy in the assessment of these relationships.

## Figures and Tables

**Table 1:** Demographic characteristics and global indices obtained in both groups.

	Glaucoma				Healthy				*P* values
	Mean	SD	Max	Min	Mean	SD	Max	Min	
Age (years)	68.43	10.93	85	35	47.93	19.24	78	24	<0.001
VFI^a^	80.84	16.24	98.00	27.00	99.0	0.95	100	96	<0.001
MD^b^	−7.73	5.58	−0.74	−23.28	−1.083	1.32	1.72	−3.39	<0.001
ATT^c^	269.75	12.77	297.00	250.00	290.00	16.77	329.00	254.00	<0.001
ATS^d^	274.34	15.02	309.00	252.00	290.68	17.46	332.00	251.00	<0.001
ATI^e^	265.39	13.81	292.00	244.00	291.10	16.30	326.00	257.00	<0.001

^a^Visual field index. ^b^Mean deviation. ^c^Total average thickness. ^d^Superior average thickness. ^e^Inferior average thickness.
